# Recent Progress in Lectin-Based Biosensors

**DOI:** 10.3390/ma8125478

**Published:** 2015-12-09

**Authors:** Baozhen Wang, Jun-ichi Anzai

**Affiliations:** 1 Department of Nutrition and Food Hygiene, School of Public Health, Shandong University, 44 Wenhua Xilu, Jinan 250012, China; Pauline_111@m.tohoku.ac.jp; 2 Graduate School of Pharmaceutical Sciences, Tohoku University, Aramaki, Aoba-ku, Sendai 980-8578, Japan

**Keywords:** lectin, biosensor, concanavalin A, glucose sensor, pathogenic bacteria, cancer cells

## Abstract

This article reviews recent progress in the development of lectin-based biosensors used for the determination of glucose, pathogenic bacteria and toxins, cancer cells, and lectins. Lectin proteins have been widely used for the construction of optical and electrochemical biosensors by exploiting the specific binding affinity to carbohydrates. Among lectin proteins, concanavalin A (Con A) is most frequently used for this purpose as glucose- and mannose-selective lectin. Con A is useful for immobilizing enzymes including glucose oxidase (GOx) and horseradish peroxidase (HRP) on the surface of a solid support to construct glucose and hydrogen peroxide sensors, because these enzymes are covered with intrinsic hydrocarbon chains. Con A-modified electrodes can be used as biosensors sensitive to glucose, cancer cells, and pathogenic bacteria covered with hydrocarbon chains. The target substrates are selectively adsorbed to the surface of Con A-modified electrodes through strong affinity of Con A to hydrocarbon chains. A recent topic in the development of lectin-based biosensors is a successful use of nanomaterials, such as metal nanoparticles and carbon nanotubes, for amplifying output signals of the sensors. In addition, lectin-based biosensors are useful for studying glycan expression on living cells.

## 1. Introduction

Biosensors are fabricated by combining molecular recognition elements, such as enzymes and antibodies, and electrical or optical transducers. Recognition elements are immobilized on the surface of transducers, resulting in reagentless sensors that can be used for determining target molecules without adding reagents in the sample solution. Immobilization of recognition elements without a loss of the biological activity is a prerequisite for successful preparation of any biosensors. Thus, immobilization of recognition elements on the surface of transducers is a key step in constructing high-performance biosensors. Therefore, a variety of protocols have been developed for immobilizing proteins on a solid surface, including irreversible adsorption through hydrophobic and electrostatic forces, chemical cross-linking with divalent reagents, entrapment in polymer networks, and covalent bonding [1,2,3]. 

Another protocol for protein immobilization is to use binding proteins as molecular glue that adheres to enzymes and antibodies through biological interactions. Typical examples of such binding proteins include antibody, avidin, and lectin. Antibodies have been widely used to immobilize antigen-tagged proteins [4,5]. Avidin is a glyco-protein isolated from egg white and known to strongly bind biotin or biotin-tagged molecules [6,7]. A variety of biotin-tagged biomolecules have been immobilized on solid surfaces to construct protein architectures and biosensors [8,9,10,11,12,13]. On the other hand, lectin is a family of sugar-binding proteins found in plants and animals [14]. Concanavalin A (Con A) is a typical lectin protein that has been most widely utilized for constructing biosensors. Con A contains four binding sites to sugars such as D-glucose and D-mannose; the binding constants to D-glucose and D-mannose are 0.8 × 10^3^ and 2.2 × 10^3^ M^−1^, respectively [15,16,17]. Early works demonstrated that polysaccharides and proteins can be immobilized using Con A onto solid supports without loss of the biological activity [18,19,20]. An advantage of using Con A in constructing biosensors is that glycoenzymes, such as glucose oxidase (GOx) and horseradish peroxidase (HRP), can be immobilized without labeling because these enzymes intrinsically contain hydrocarbon chains [21,22,23,24,25].

**Figure 1:**
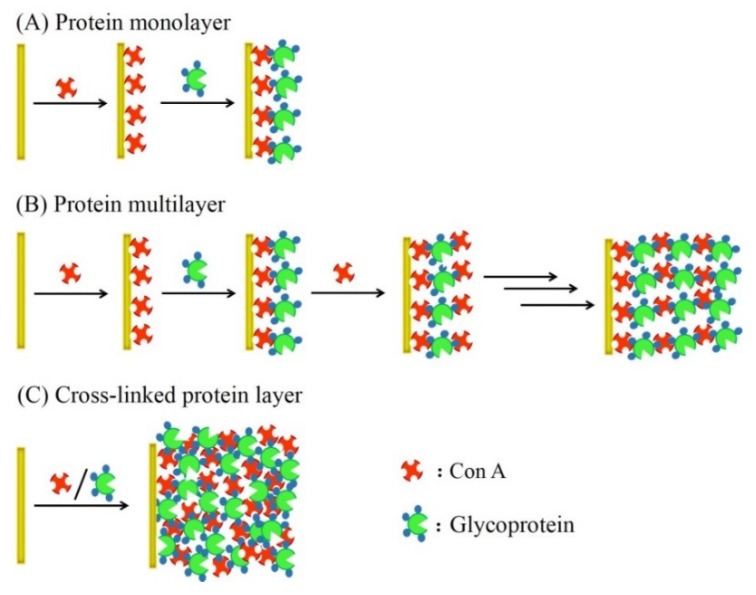
Possible routes for immobilization of glycoproteins by Con A on the surface of solid surface. (**A**) Monomolecular layer of glycoprotein; (**B**) layer-by-layer-deposited multilayer film of glycoprotein; and (**C**) cross-linked multilayer film of glycoprotein.

Three different routes are available for immobilizing proteins on the surface of transducers by means of Con A. Glycoproteins or sugar-labeled proteins can be deposited on Con A-adsorbed transducers by simply immersing the transducers in the solution of proteins (Figure 1a). This procedure provides basically a monomolecular layer of the proteins. The amount of immobilized proteins can be enhanced by forming multilayers, in which Con A and proteins are alternately deposited on the surface of transducers in a layer-by-layer (LbL) fashion (Figure 1b). In this protocol, the amount of immobilized proteins depends on the number of layers, enabling precise control of the magnitude of sensor signals. LbL-deposited protein films have recently attracted much attention because of their potential applications to controlled release and biosensors [26,27,28]. Another protocol relies on cross-linking of glycoproteins with Con A in the mixed solution to form a protein gel layer on the transducer surface (Figure 1c).

Lectins are used not only as molecular glue to immobilize proteins but also used as the recognition element of biosensors, because lectins bind carbohydrate chains with high selectivity [29,30,31,32]. Thus, lectin-modified electrodes have been used as sensors for the determination of glucose, glycoproteins, as well as cells and microorganisms. 

The use of lectins in the development of sugar-sensitive materials for biosensors has been attracting much attention [33,34,35,36,37]. Many different biosensors have been developed using lectin-modified electrodes and optical devices. Therefore, in this review, we focus on progress in the development of lectin-based biosensors in the last several years. Biosensors for the determination of glucose, pathogenic bacteria and toxins, cancer cells, and lectins are discussed.

## 2. Glucose Sensors

The development of GOx-based glucose sensors has been a focal subject in the field of biosensors [38]. On the other hand, metal and carbon nanomaterials are employed for constructing non-enzymatic glucose sensors [39]. Organic materials such as conducting polymers and phenylboronic acids are also used for developing enzyme-free glucose sensors [40,41,42,43]. In this context, early studies demonstrated the utility of Con A in constructing optical and electrochemical glucose sensors [44,45,46,47,48,49,50,51]. For example, Con A was used for immobilizing GOx on the surface of electrodes. In contrast, in non-enzymatic glucose sensors, Con A itself serves as a recognition element for glucose. 

A typical example of GOx-based glucose sensor has been prepared by mixing HRP-GOx conjugate, Con A, and chitosan (CS) on the surface of a gold (Au) electrode to form cross-linked HRP-GOx-Con A-CS thin films [52]. The HRP-GOx-Con A-CS films were rather porous and the GOx was catalytic active in the films. Au electrodes modified with the film exhibited amperometric response to glucose over the concentration range of 1.0 × 10^−6^–2.2 × 10^−4^ M with a lower detection limit of 6.7 × 10^−^^7^ M. The response of the sensor was satisfactorily fast (~10 s) owing to high porosity of the HRP-GOx-Con A-CS film. A variety of glucose sensors were constructed by immobilizing GOx through Con A linkages on Au/platinum (Pt) hybrid nanostructures [53], carbon nanotube-supported Pt nanoparticles [54], and Prussian blue/graphene composite films [55]. The linear response ranges of the glucose sensors were 3.0 × 10^−^^6^–2.3 × 10^−^^3^ M [53], 1.2 × 10^−^^6^–2.0 × 10^−^^3^ M [54], and 2.5 × 10^−^^5^–3.2 × 10^−^^3^ M [55], respectively. The lower detection limits of the sensors were in the range of 4.0 × 10^−^^7^–1.0 × 10^−^^5^ M. Zinc oxide nanorods modified with Au/Pt composite were also combined with GOx-Con A layers to fabricate glucose sensors, which exhibited a response to glucose from 1.8 × 10^−^^6^ to 5.1 × 10^−^^3^ M and a lower detection limit was 0.6 × 10^−^^6^ M [56]. Bienzyme sensors consisting of GOx and HRP were prepared based on LbL deposition of Con A and enzymes on the surface of electrode and the electrochemical response to phenols, aromatic amines, and sulfides was studied [57,58]. 

**Figure 2:**
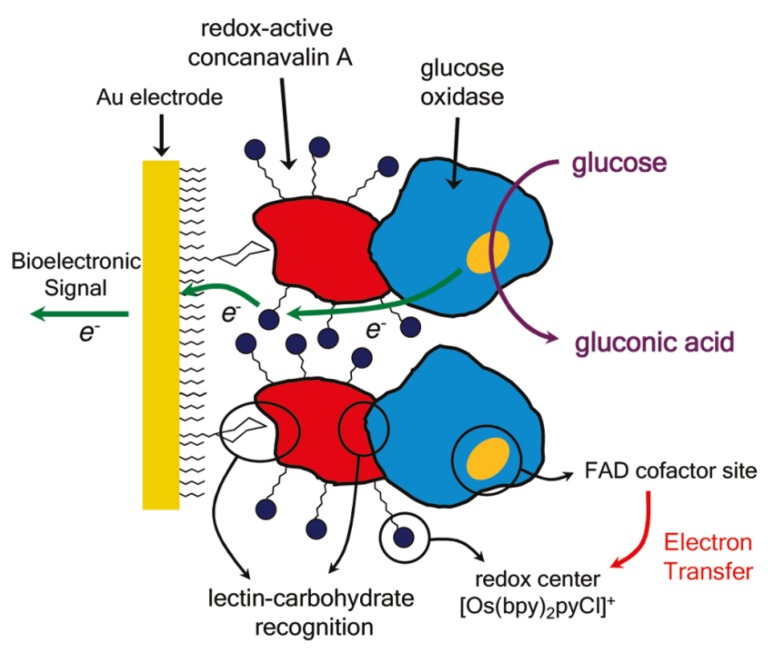
A glucose sensor based on redox-active Con A and GOx. Reprinted with permission from Langmuir. Copyright 2010 American Chemical Society [59].

GOx-based electrochemical glucose sensors are often operated in the presence of electron transfer mediators, such as metal complexes, that can shuttle electrons from reduced form of GOx to electrode. The electron transfer mediators are co-immobilized on the surface of electrodes together with GOx, providing mediator-type glucose sensors. On this line, mediator-type glucose sensors can be constructed using redox-active Con A modified with electron transfer mediators for immobilizing GOx on electrode. According to this concept, Azzaroni and Battaglini groups have recently constructed biosensor platforms using redox-active Con A modified with osmium bipyridine complex, [Os(bpy)_2_Clpy] [59,60,61,62]. The [Os(bpy)_2_Clpy]-modified Con A was used to immobilize GOx on the surface of Au electrode (Figure 2). Electrochemical and optical studies showed that [Os(bpy)_2_Clpy]-modified Con A successfully accelerates electron transfer from GOx to electrode, although only a fraction (*i.e*., 20%–30%) of the redox sites is involved in the electron transfer. The same group showed that HRP can be assembled together with GOx using [Os(bpy)_2_Clpy]-modified Con A to provide bienzyme glucose sensors [60]. In another study, redox-active Con A bearing daunomycin was prepared to evaluate interactions between Con A and ovalbumin [63]. The redox response of the daunomycin-labeled Con A decreased in the presence of 1.5 × 10^−^^1^^0^ to 1.5 × 10^−^^9^ M ovalbumin as a result of specific binding of ovalbumin to Con A. Nanomaterials such as graphene oxide sheets [64] and aluminum oxide particles [65] were successfully coupled with Con A to provide support for enzyme immobilization. pH and thermal stability of enzymes were improved by using the nanomaterials as support. Apart from Con A-mediated glucose sensors, HRP-modified electrodes were prepared through Con A complexation to study electron transfer reactions between HRP and electrode [66,67,68]. 

**Figure 3:**
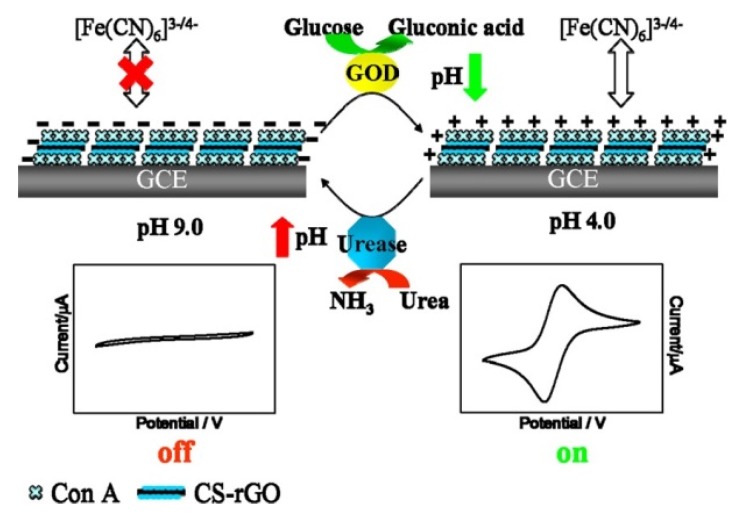
Con A-coated glassy carbon electrode as a pH-sensitive ion gate for glucose and urea sensing. Reprinted with permission from Analytical Chemistry. Copyright 2014 American Chemical Society [72].

Con A can be used as glucose recognition element for the construction of non-enzyme glucose sensors by taking advantages of glucose selectivity of Con A. Glucose sensors have been constructed based on a variety of signal transduction mechanisms, including electrochemistry, colorimetry, fluorometry, and gravimetry. For example, capacitive glucose sensors were constructed by immobilizing Con A on a poly(tyramine)/Au nanoparticles-coated electrode [69]. The capacitive glucose sensors showed a response to 1.0 × 10^−^^6^–1.0 × 10^−^^2^ M glucose, though the response was rather slow (*ca.* 15 min). Amperometric sensors using thionine-modified electrodes were also reported [70,71]. The thionine-modified electrodes exhibited responses to glucose in the ranges of 1.0 × 10^−^^6^–1.0 × 10^−^^4^ M with a detection limit of 7.5 × 10^−^^7^ M [70] and 5.0 × 10^−^^7^–1.55 × 10^−^^5^ M with a detection limit of 2.2 × 10^−^^7^ M [71]. In another example, surface-confined Con A served as a pH-sensitive ion-gate that blocks redox-active ions depending on pH of the solution [72] (Figure 3). Thus, voltammetric signal of the Con A-confined electrode depends on GOx-catalyzed reaction of 1–10 mM glucose in solution, because the enzymatic reaction produces an acidic product, *i.e.*, gluconic acid. Urea sensors can also be constructed using this system by combining with urease. A drawback of this system is that redox-active species, ferricyanide ions, have to be added in the sample solution. 

Mannose-coated Au nanoparticles were used to develop a colorimetric sensor for glucose [73]. The addition of Con A in the Au nanoparticles dispersion induced aggregation of the Au nanoparticles as a result of cross-linking by Con A, whereas aggregation was suppressed in the presence of glucose due to the competitive binding of glucose to Con A. The aggregated Au nanoparticles were detected through color changes which originated from the shift of surface plasmon absorption band. The colorimetric sensor detected glucose in human serum in the range of 500–4000 μg/mL within 10 min with a lower detection limit of 363 μg/mL. In a similar strategy, fluorescence resonance energy transfer (FRET) between two quantum dots modified with Con A and glucose was studied in the presence of glucose [74]. A needle-type optical biosensor for continuous glucose monitoring was fabricated by combining an optical fiber probe with syringe needles [75]. The optical fiber probe was modified with a thin film composed of Con A and mannose-modified bovine serum albumin (m-BSA). The layer thickness of the m-BSA/Con A conjugate depends on the concentration of glucose in sample solution as a result of the competitive binding of glucose to Con A, inducing a shift in the wavelength of the guided light. The sensor was able to monitor the concentration of glucose in blood in the range of 10–500 mg/dL with response time of 15 min. This sensor may be promising for clinical applications because the detection range covers the physiological relevant level of blood glucose. 

A quartz crystal microbalance (QCM) has been used to develop gravimetric glucose sensors by immobilizing dextran/Con A conjugates on the surface of quartz resonator [76]. The dextran/Con A conjugates decompose upon exposing the resonator to glucose, resulting in changes in the resonance frequency of QCM. Equilibrium constants in the binding of Con A and saccharides were determined by recording changes in the resonance frequency associated with the binding and release of saccharides on Con A-modified quartz resonator [77]. In addition, silicon nanowire-based field-effect transistors [78] and surface plasmon resonance (SPR) sensors [79] were also used for the evaluation of carbohydrate-lectin interactions. 

## 3. Pathogenic Bacteria and Toxin Sensors

The detection of pathogenic bacteria is a prerequisite for food and water safety, disease diagnostics, process control in food and pharmaceutical industries, and so forth. Conventional methods for bacteria detection, such as culture and colony counting [80] and enzyme-linked immunosorbent assay [81] are somewhat laborious and time-consuming. Consequently, alternative methods are required for the rapid and reliable detection of bacteria. For this goal, use of lectin-modified sensors may be a promising approach because carbohydrate chains are often expressed on the surface of bacteria. 

**Figure 4:**
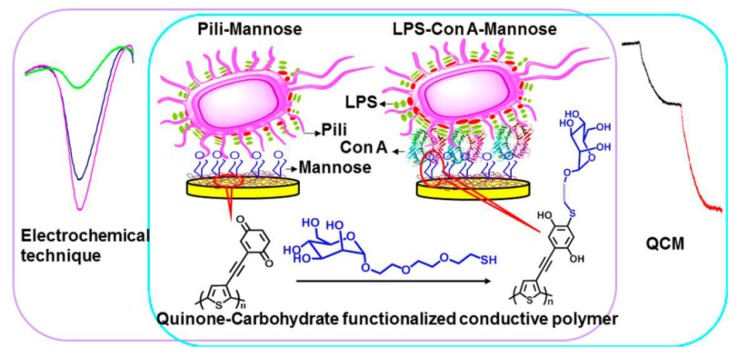
An *E. coli* sensor based on a quinone-fused poly(thiophene) film-coated electrode and its electrochemical and gravimetric responses. Reprinted with permission from Analytical Chemistry. Copyright 2015 American Chemical Society [84].

Several groups have reported lectin-based bacteria sensors. Pingarrón and coworkers developed electrochemical impedance sensors for the detection of *Escherichia coli* (*E. coli*) using screen-printed Au electrodes [82]. Con A was added to sample solutions containing *E. coli* to form *E. coli*-Con A aggregates, which were then adsorbed to the electrode. The electron transfer resistance of the electrode linearly depended on the logarithmic concentration of *E. coli* over 5.0 × 10^3^–5.0 × 10^7^ colony forming units (cfu)/mL. Interestingly, bacteria adsorption to the electrode was negligible without Con A. The authors evaluated the electrochemical responses of the sensor to aggregates formed between nine kinds of lectins and three different bacteria, *i.e.*, *E. coli*, *Staphilococcus aureus* (*S. aureus*), *and Mycobacterium phlei.* Principal component analysis allowed classification of the bacteria. Another study also used electrochemical impedance spectroscopy for the detection of *E. coli*, in which antibodies were used as *E. coli*-recognition elements in place of Con A [83]. An *E. coli* sensor based on a quinone-fused poly(thiophene)-coated Au electrode has recently been reported [84]. The surface of the Au electrode was coated with electrochemically-polymerized poly(thiophene) film, followed by substitution with mannose residues (Figure 4). *E. coli* was captured on the electrode through two different routes, *i.e.*, a direct binding and Con A-mediated binding. This sensor can be operated both in electrochemical and gravimetric modes. In the electrochemical measurement, the peak current in square wave voltammetry of the sensor decreased with increasing the concentration of *E. coli* owing to the disturbed electron transfer of quinone-fused thiophene moiety. In the QCM detection mode, on the other hand, the resonance frequency of the sensor changed depending on *E. coli* concentration. The lower detection limits of the sensor were 25 and 50 cells/mL in the electrochemical and QCM detections, respectively. Ferrocene-tagged boroxanol pentamer (Fc-pentaBZE) was synthesized by Scheller and coworkers for the development of electrochemical displacement sensors for *E. coli* [85]. Fc-pentaBZE was immobilized onto a mannose monolayer-coated electrode as a redox-active marker. It is known that boroxanol and boronic acids covalently bind diol compounds such as sugars by forming boronate ester linkage [86]. The immobilized Fc-pentaBZE was displaced by *E. coli* or Con A upon immersing the electrode in the solution containing *E. coli* or Con A, resulting in the changes in redox signals of Fc-pentaBZE. The lower detection limit of this sensor for *E. coli* was approximately 6 × 10^2^ cells/mL. In this context, use of synthetic lectins such as boroxanol- and boronic acid-modified proteins and polymers may be interesting [87,88,89,90,91]. These materials are endowed with sugar-binding ability because boronic acids form cyclic ester bonds with 1,2- and 1,3-diol compounds including sugars. A variety of synthetic lectins have been used for constructing sugar-sensitive systems [92,93,94,95,96,97]. Future applications of synthetic lectins to biosensors would be promising.

A wireless magnetoelastic sensor for *E. coli* O157:H7 was developed based on Con A- and chitosan-modified magnetic Fe_3_O_4_ nanoparticles [98,99]. An advantage of magnetoelestic sensors is that no physical contact between the sensor probe and the detection system is needed because the sensors require no internal power source. For constructing Con A-mediated sensors, Fe_3_O_4_ nanoparticles were modified with mannose and Con A simultaneously. The resonance frequency of the probe changed upon binding *E. coli*, enabling determination of *E. coli* concentration. The sensor exhibited a linear response to *E. coli* over the logarithmic concentration of 6.0 × 10–6.1 × 10^9^ cells/mL. Likewise, a variety of *E. coli* sensors were developed using lectins on the basis of colorimetry [100], flow cytometry [101], and microscopy [102]. 

Bacterial toxins can be detected through lectin complexation because bacterial toxins often contain sugar moiety as a component. For example, lipopolysaccharides (LPS) located on the outer membrane of Gram-negative bacteria and lipoteichoic acid (LTA), which is composed of glycerolphosphate and saccharides, found in Gram-positive bacteria have been made subject to lectin-mediated detection. Andrade and coworkers developed electrochemical sensors sensitive to LPS from *E. coli* and LTA from *S. aureus* [103]. They prepared Con A/poly(aniline) film-coated electrodes and their impedimetric response was recorded in the presence of bacterial toxins. The charge transfer resistance of the Con A-modified electrode significantly increased upon binding the bacterial toxins in the range of 50–200 μg/mL. On the other hand, LPS sensors prepared using Con A-confined electrodes were used for the assay of activity of antibiotics [104]. The response of the Con A-modified sensor to *E. coli* was monitored in the presence of antibiotics. The output signals originating from LPS of *E. coli* reduced to 23%, 27% and 38% after 18-h incubation of *E. coli* in the presence of ciprofloxacin, ceftriaxone, and tetracycline, respectively. Thus, Con A-modified sensors are useful for evaluating the antibiotic activity of drugs.

The interactions between hemagglutinin (HA) from human influenza virus and several affinity ligands were studied using surface plasmon resonance (SPR) sensors for screening suitable ligands for the development of influenza virus sensors [105]. HA is an immunogenic glycoprotein found on the surface of influenza virus. Lectins such as wheat germ agglutinin (WGA), *Maackia amurensis* lectin (MAL), *Sambucus nigra* agglutinin (SNA), and Con A as well as sialic acid derivatives were tested as candidates for the ligand. Among them, 6’-sialyllactose-ovabumin conjugate was best suited for constructing influenza virus sensors. The SPR sensors were successfully used for analyzing HA in the range of 10–100 μg/mL. It is also possible to use metal-clad waveguide sensors, which rely on refractivity changes, for constructing Con A-mediated biosensors [106]. Potential use of the waveguide sensors for label-free measurement of the viability of animal cells, which were attached to the surface of sensor probe through Con A, was suggested.

Fe_3_O_4_/MnO_2_ nanoparticles modified with ferrocene (Fc) and Con A were used for enhancing the output signals of electrochemical bacteria sensors [107]. Target bacteria, *Deslforibrio caledoiensis (D. caledoiensis)*, were adsorbed to Con A-immobilized glassy carbon electrode and the electrode was incubated in the solution of Fc/Con A-modified Fe_3_O_4_/MnO_2_ nanoparticles. The electrochemical response of the electrode increased with increasing the *D. caledoiensis* concentration in the range of 1.0 × 10^3^–1.0 × 10^8^ cfu/mL. Use of Fc as electrochemical redox marker is a promising approach to develop highly sensitive amperometric and voltammetric biosensors [108]. 

**Figure 5:**
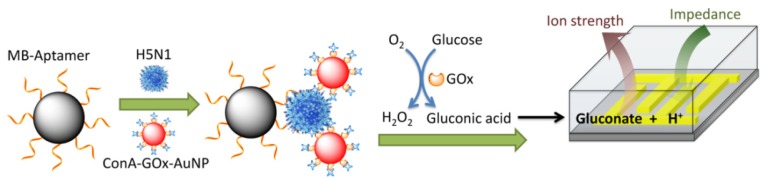
Avian influenza virus sensing based on nano-composites consisting of GOx, Con A, and Au nanoparticles. Reprinted with permission from Analtical Chemistry. Copyright 2014 American Chemical Society [112].

Electrochemical impedance sensors sensitive to serum proteins from patients infected with dengue virus have been reported [109,110,111]. The sensor was prepared by coating the surface of Au electrode with a lipid membrane doped with Con A. The sensors exhibited different responses to four serotypes of dengue virus, resulting in the classification of the type of dengue virus. An interesting protocol for impedimetric sensing of avian influenza virus was developed using magnetic beads modified with avian influenza virus H5N1-specific aptamer (Figure 5) [112]. The modified beads were used for binding influenza virus, followed by conjugation with Con A, GOx, and Au nanoparticles to provide a nanocomposite. The nanocomposite was transferred to a glucose solution to induce GOx-catalyzed oxidation reaction of glucose, resulting in the enhancement of ionic strength due to enzymatically-produced gluconic acid. Thus, impedance of an interdigitated array electrode decreased depending on the amount of GOx on the nanocomposite which, in turn, depends on the concentration of influenza virus in the sample solution. This sensor exhibited a linear response to avian influenza virus H5N1 in the logarithmic concentration of 1.0 × 10^−3^–1.0 × 10^0^ hemagglutination units (hau) with a detection limit of 8.0 × 10^−4^ hau. A merit of this protocol is that only an unmodified electrode is required for measuring the electrochemical impedance and the nanocomposites can be prepared separately. Other glycoproteins such as transferrin and immunoglobulin were also made subject to electrochemical determination on Con A-modified electrodes [113,114,115].

## 4. Cytosensors

Cytosensors, *i.e.*, sensors for detecting cells, have been constructed using lectin-modified electrodes by exploiting selective binding of lectins to hydrocarbon chains expressed on the surface of cells. Target cells are bound to the lectin-modified cytosensors to induce changes in electrochemical signals, depending on the number of cells in the sample solutions. Therefore, cytosensors can be used for the quantitative determination of cells. In addition, cytosensors are useful for the profiling of hydrocarbon chains expressed on cell surface because binding affinity of lectins significantly depends on the type of components of hydrocarbon chains. 

**Figure 6:**
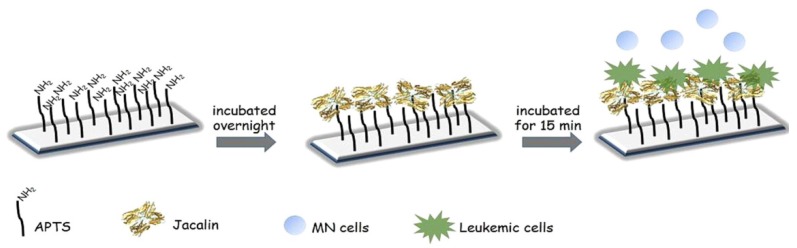
Preparation of impedimetric cytosensors based on a jacalin-modified electrode for the determination of cancer cells. Reprinted with permission from ChemElectroChem. Copyright 2015 Wiley-VCH Verlag GmbH and Co. [119].

Recently, much attention has been devoted to the important role of glycoproteins and glycolipids on the surface of cancer cells in relation to cancer therapeutics and diagnosis [116]. In this context, lectin-modified electrodes have been studied to develop cytosensors for detecting cancer cells. Electrochemical cytosensors were constructed using Con A-modified Au electrode coupled with Au nanoparticles coated with Con A and ferrocenylhexanethiol (Fc-SH) [117]. The Con A-modified electrode was first incubated in the sample solution to bind target cancer cells (K562 leukemic cells), followed by a further deposition of the Au nanoparticles to enhance output signal of the sensor. The voltammetric response of the sensor, which originates from redox reactions of Fc moieties, depended on the cell concentration in the range of 1.0 × 10^2^–1.0 × 10^7^ cells/mL. Con A-modified Au electrodes can be used for impedimetric detection of cancer cells [118]. The charge transfer resistance of the sensor was dependent on the number of human liver cancer cell Bel-7404 in sample solution. The lower detection limit of the sensor was 234 cells/mL. Zucolotto and coworkers used a galactose-selective lectin jacalin for constructing impedimetric sensors for monocytic leukemic cells THP-1 and myeloblastic cells OCl-AMI3 [119] (Figure 6). The jacalin-based sensors differentiated leukemic cells from healthy monocyte cells with detection limits of 3 ± 1 cells/mL for THP-1 and 4 ± 1 cells/mL for OCl-AMI3. Furthermore, QCM sensor was useful for evaluating changes of carbohydrate chains of cancer cells associated with tumor growth and metastasis. In fact, Pei and coworkers immobilized an epidermoid carcinoma cell line (A-4531) and a breast adenocarcinoma cell line (MDA-MB-468) on quartz crystals to evaluate the binding of lectins [120]. The gravimetric response of the QCM sensors was sensitive to the glycosylation changes of the cells associated with cancer progression and development. 

**Figure 7:**
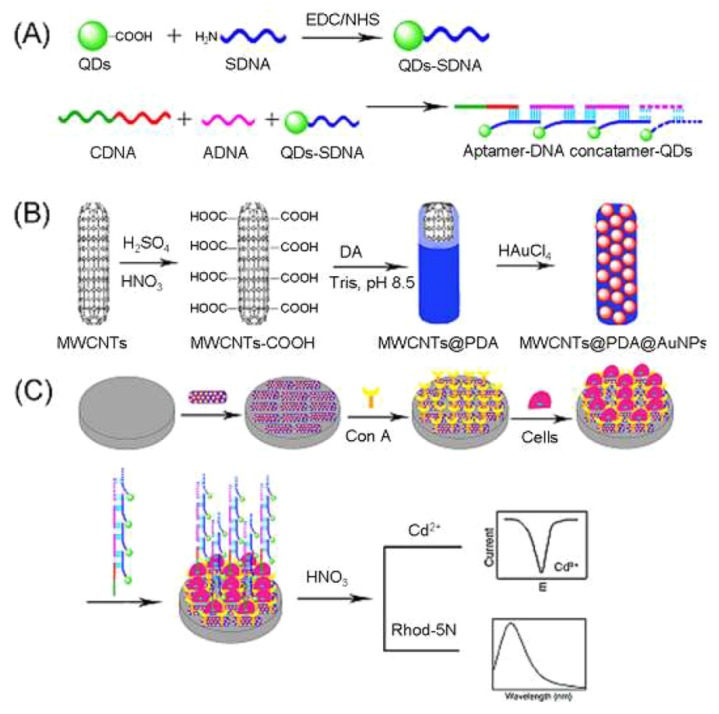
Preparation of nanocomposite-based cytosensors. Precedures for (**A**) preparing aptamer-DNA concatamer quantum dots by coupling of quantum dot and single-strand DNA followed by hybridization with aptamer and complementary DNAs; (**B**) preparing Au nanoparticles-modified carbon nanotubes by coating with poly(dopamine) film and Au deposition; and (**C**) preparing nanocomposite-modified electrodes by successive deposition of Au nanoparticles-modified carbon nanotubes, Con A, and target cells, and aptamer-quantum dots composites on the surface of a glassy carbon electrode. The electrode was immersed in HNO_3_ solution to dissolve the residual quantum dots and then the concentration of cells was determined with electrochemical or fluorescence method. Reprinted with permission from Analytical Chemistry. Copyright 2013 American Chemical Society [121].

A strategy for signal amplification in the detection of cancer cells was proposed based on Con A-modified composite materials consisting of carbon nanotubes and Au nanoparticles combined with quantum dots [121] (Figure 7). The sensor showed an electrochemical response to model cancer cells (CCRF-CEM cells) in the range of 1.0 × 10^2^–1.0 × 10^6^ cells/mL and the detection limit was 50 cells/mL. The response range and detection limit of the sensor in fluorescence response was almost same to those of the electrochemical response. A key role of the aptamer-DNA concatamer quantum dots in the highly-sensitive determination of the cancer cells was suggested. 

Nanomaterial-based cytosensors for the detection of human lung cancer cells, 95-D and H1299, was reported [122]. The cytosensor was constructed by depositing polymer-coated carbon nanotubes, glutathion-protected Au nanoparticles, and Con A successively on the surface of a glassy carbon electrode. The sensor was exposed to a sample solution containing target cancer cells and mannose/thionine-modified nanoparticles, in which nanoparticles and cancer cells competitively bind to Con A on the sensor surface. Voltammetric response of the sensor, originating from redox reactions of thionine, was reversely dependent on the concentration of cancer cells in the sample solution. The sensor showed linear calibration graphs for the logarithmic concentrations ranging from 1.7 × 10^3^ to 1.5 × 10^8^ cells/mL for 95-D with a lower detection limit of 580 cells/mL and from 2.5 × 10 to 1.0 × 10^6^ cells/mL for H1299 with the detection limit of 12 cells/mL. In a similar strategy using Au nanoparticle/quantum dot composites, A549 lung cancer cells and QGY-7701 liver cancer cells in the concentrations ranging from 10 to 10^7^ cells/mL and from 10^4^ to 10^7^ cells/mL, respectively, were detected based on the fluorescence emission of the quantum dots [123]. Other nanomaterials useful for enhancing the response of lectin-based cytosensors include nanocomposites comprising Ru(bpy)_3_^2+^-doped Au/silica nanoparticles [124], HRP/aptamer-modified Au nanoparticles [125], protein-modified silver nanoflowers [126], and GOx-modified Au nanoparticles [127]. Li and coworkers demonstrated that chemiluminescence biosensors based on the Ru(bpy)_3_^2+^-doped Au/silica nanoparticles can be used for detecting K562 cells in the range of 1.0 × 10^3^–1.0 × 10^7^ cells/mL with a detection limit of 600 cells/mL [124]. The same group further improved the detection limit of human acute lymphoblastic leukemia cells down to 10 cells/mL [125] and that of K562 cells to 18 cells/mL [127]. On the other hand, silver nanoflower-based cytosensors detected human colon cancer cells (DLD-1) in the concentration ranging from 1.35 × 10^2^ to 1.35 × 10^7^ cells/mL with a detection limit of 40 cells/mL [126]. 

## 5. Lectin Sensing

**Figure 8:**
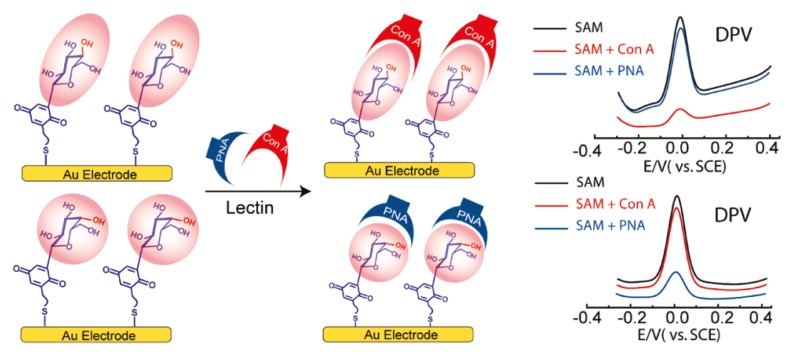
Epimeric monosaccharide-quinone hybrid monolayer-modified Au electrodes for lectin sensing. Reprinted with permission from Journal of the American Chemical Chemistry. Copyright 2011 American Chemical Society [128].

Biosensors sensitive to lectin can be prepared by using carbohydrates as recognition elements. In fact, a variety of devices have been developed by immobilizing carbohydrate chains on the surface of electrode and other transducers. A prototype of lectin sensor was constructed by modifying the surface of Au electrode with monosaccharide-quinone hybrids (Figure 8) [128]. In this study, glucose-quinone and galactose-quinone hybrids were used to form monomolecular layers sensitive to specific lectins, Con A and peanut agglutinin (PNA), respectively. The voltammetric signals of the sensors arising from quinone moieties significantly decreased upon binding specific lectin, whereas the response to non-specific lectin was negligible. The results clearly demonstrated that specific epimeric sugars, *i.e.*, glucose or galactose, are discriminated by lectin on the sensor. Au film-coated quartz crystal probes coated with a mannose-quinone functionalized poly(thiophene) film was likewise used to construct lectin sensors [129]. This sensor can be operated in electrochemical as well as gravimetric (*i.e*., QCM) detection modes. The sensor exhibited dynamic ranges of 0.5–17.5 nM in the electrochemical mode and 0.5–4.5 nM in the QCM mode, respectively, in the detection of Con A.

A variety of materials have been used to construct lectin sensors, including self-assemblies of glucose and mannose derivatives [130,131], glycosylated poly(aniline)s [132,133], and glycolipid vesicles [134]. Among the above sensors, it is noteworthy that the lower detection limit of poly(aniline)-based Con A sensor was highly improved, *i.e.*, 1.0 × 10^−12^ M [132]. Lectin-induced decomposition reaction of redox-active daunomycin-doped micells was also used for the detection of Con A in the range of 2.0 × 10^−9^–8.0 × 10^−8^ M [135]. 

Electro-chemiluminescence (ECL)-based lectin sensing was reported by Chen and coworkers [136,137,138]. The ECL-based system was constructed using nanocomposites comprising graphene, Au nanoparticles, and GOx, in which ECL signal significantly decreased upon binding Con A. Thus, the ECL-based sensors could detect Con A in the range of 0.1–100 ng/mL [136], 1.0–20 ng/mL [137], and 0.05–100 ng/mL [138]. In addition, fluorescence-based systems sensitive to lectins were constructed based on carbohydrate polymers [139] and carbohydrate-capped Au nanoparticles [140]. The carbohydrate polymers and carbohydrate-capped Au nanoparticles could be used for detecting Con A in the ranges of 1–250 nM [139] and 0.1–500 μg/mL [136], respectively. These materials would be useful for constructing optical sensors if they are combined with appropriate optical devices. 

## 6. Conclusions

Biosensors can be constructed through selective complexation between lectins and carbohydrate chains. The usefulness of the lectin-carbohydrate affinity for constructing biosensors is that any sugar-tagged proteins can be immobilized on the surface of electrochemical and optical transducers. Furthermore, some enzymes, such as GOx and HRP, can be used directly to construct biosensors without labeling owing to their intrinsic hydrocarbon chains. It is an advantage of lectin-carbohydrate systems that the whole process of enzyme immobilization can be carried out under mild aqueous conditions. A versatility of lectins in the recognition of carbohydrates provides another advantage. In other words, lectins are used not only as molecular glue for protein immobilization but lectins serve as recognition element of biosensors. In fact, Con A-modified electrodes are used to detect pathogenic bacteria and cancer cells, in which Con A selectively binds carbohydrate chains located on the surfaces of bacteria and cancer cells. One of the recent trends in the development of lectin-based biosensors is a successful use of metal and carbon nanomaterials for amplifying the output signals of sensors. The nanomaterials make it possible to accumulate significant amounts of optically- and electrochemically-active compounds on the sensors, enabling signal amplification. Au nanoparticles and carbon nanotubes are used for this purpose.
